# Acceptability and Palatability of Novel Orodispersible Minitablets of Enalapril in Children up to the Age of 6 with Heart Failure

**DOI:** 10.3390/jcm14030915

**Published:** 2025-01-30

**Authors:** Milica Lazic, Milan Djukic, Vladislav Vukomanovic, Maja Bijelic, Emina Obarcanin, Milica Bajcetic

**Affiliations:** 1 Institute of Pharmacology, Clinical Pharmacology and Toxicology, Faculty of Medicine, University of Belgrade, 11129 Belgrade, Serbia; milicalazic272@gmail.com; 2 Department of Cardiology, University Children’s Hospital, 11129 Belgrade, Serbia; milandjukic62@gmail.com (M.D.); bijelicmaja11@yahoo.com (M.B.); 3 Department of Pediatrics, Faculty of Medicine, University of Belgrade, 11129 Belgrade, Serbia; vvukomanovicdr@gmail.com; 4 Department of Cardiology, Institute of Mother and Child Health “Dr Vukan Čupić”, 1070 Belgrade, Serbia; 5 Institute of Clinical Pharmacy and Pharmacotherapy, Heinrich-Heine-Universitaet Düsseldorf, 40225 Duesseldorf, Germany; emina.obarcanin@uni-duesseldorf.de

**Keywords:** heart failure, pediatric cardiology, enalapril, acceptability, palatability, pediatric drug formulations, infants and toddlers

## Abstract

**Background:** Angiotensin-converting enzyme inhibitors, such as enalapril, are foundational in treating pediatric heart failure. However, they are often administered off-label to young children using extemporaneous formulations. This study, conducted as part of the EU-funded Labeling of Enalapril from Neonates up to Adolescents (LENA) project, aimed to evaluate the acceptability and palatability of an age-appropriate enalapril orodispersible minitablet (ODMT). These factors are critical for ensuring adherence, efficacy, and safety in pediatric patients. **Methods:** An 8-week trial was conducted in children with heart failure caused by dilated cardiomyopathy or congenital heart disease. Enalapril ODMTs (0.25 mg or 1.0 mg) were dose-titrated and administered to 38 children aged 0–6 months and 22 children aged 6 months to 6 years. This study aimed to assess its acceptability and palatability, key factors contributing to adherence, and therefore, efficacy and safety. **Results:** Across all 169 assessments in 38 children aged 0–6 months and 22 aged 6 months to 6 years, complete or partial swallowability was observed, and the acceptability rate was 100%. There were no cases of choking, inhalation/coughing, or spitting out. A favorable or neutral rating was observed in 96% of palatability assessments based on observations of facial expressions. Acceptability and palatability were higher in subjects aged 6 months–6 years than 0–6 months, with no significant influence from repeated administration. **Conclusions:** Enalapril ODMTs are widely accepted and well-tolerated among young children, including neonates, with heart failure. These findings suggest that ODMTs are a suitable and effective method for administering pediatric medicinal products.

## 1. Introduction

Pediatric heart failure (HF) is a significant health issue with high morbidity, frequent hospitalizations, and substantial impact on quality of life. Management approaches involve extrapolation from adult therapies, despite differences in pathophysiology between children and adults [1,2]. This raises concerns about the adequacy of treatments, emphasizing the needs for evidence-based, personalized therapies tailored to pediatric patients. Pediatric patients respond differently to medications due to factors like weight, age, genetics, disease characteristics, and co-morbidities, emphasizing the need for adaptable, age-appropriate medications [3,4,5].

Until recently, oral liquid formulations were preferred in the pediatric population due to their ease of administration and dose flexibility although they present challenges such as stability, preservatives, and a short shelf life [6]. In contrast, solid formulations such as tablets and capsules offer stability and ease of storage but may not be suitable for younger children, who have difficulties swallowing and require precise dose adjustments [7].

A key challenge in pediatric HF therapy is the lack of commercially produced oral preparations for children under 6 years of age [8]. Oral medications available on the market are predominantly formulated for adults in forms that children cannot easily use, such as tablets and capsules. Consequently, pediatric treatment often involves the use of medications prepared in local pharmacies. The preparation involves crushing tablets or opening capsules intended for adult use, thus leading to inadequate dosing and absorption, collectively influencing the success and safety of the therapy. Such modified preparations are unlicensed medicines, unless the modification of a commercial product has been documented and approved by regulatory authorities in its summary of product characteristics (SmPC) [9,10].

Angiotensin-converting enzyme inhibitors (ACEIs), such as enalapril, are first-line treatments for pediatric HF but are often prescribed off-label, with limited data on their use in young children [10,11]. Enalapril is available in tablet form in Europe, lacking licensed age-appropriate and stable formulations. Liquid solutions are prepared by crushing and dispensing tablets, and these liquid preparations require preservation, are less portable, and are susceptible to various chemical processes, such as cyclization and hydrolysis, compromising the integrity of the drug [12].

In response to these challenges, the European Medicines Agency (EMA) and the World Health Organization (WHO) have advocated for compact, small solid dosage forms rather than liquid formulations [5,13,14,15]. Moreover, the EMA emphasizes the importance of assessing patient acceptability during development, as it impacts adherence, safety, and efficacy [13]. Acceptability is influenced by factors like age, disease type, capability, and patient state, as well as product characteristics such as palatability, swallowability, shape, and appearance. Palatability, a key determinant, includes smell, taste, aftertaste, and texture [13,16].

The challenges in ensuring access to suitable medicines for children prompted the WHO in 2008 to recommend flexible solid oral dosage (FSOD) forms as the preferred formulations for pediatric use [17]. Orodispersible formulations, such as orally disintegrating tablets (ODTs), address some of the FSODs drawbacks (such as water access and formulation costs) by rapid disintegration and dissolution in saliva without water intake [18,19,20]. This landscape has been further transformed by the development of small-sized tablets, known as minitablets. Orally disintegrating minitablets (ODMTs) merge the benefits of ODTs and minitablets and can be tailored for pediatric therapy. ODMTs, typically 1 to 3 mm in diameter, represent a recent innovation, but with limited commercial availability [21,22]. ODMTs offer precise dosing with no preparation needed and allow dose flexibility based on age and weight. They require fewer excipients and provide greater stability than liquid formulations [23].

Studies highlight the significant advantages and potential of minitablets over syrups or dispersible granules as a viable pediatric dosage form in terms of acceptability among children of different ages from neonates up to preschool children [23,24,25,26,27]. This has led to a shift toward small-sized solid dosage forms for pediatric use, including for infants. However, further data on the acceptability and palatability of these formulations, particularly those containing active ingredients and for chronic use, is needed. To address these gaps, the European Commission funded the LENA project to develop an innovative enalapril ODMT formulation for children. A recently published article demonstrated that pediatric patients with HF due to CHD treated with enalapril ODMTs for 8 weeks experienced favorable improvements in left ventricular diastolic dimension and HF symptoms [28].

This is the first study to assess the acceptability and palatability of a novel enalapril ODMT in pediatric patients. The evaluation was conducted across various dosage regimens, including both single and multiple doses, tailored to individual patient requirements. Our study aimed to provide a detailed assessment of the acceptability and palatability of this formulation in children ranging from birth to 6 years of age, addressing a critical gap in the literature on pediatric cardiovascular pharmacotherapy.

## 2. Materials and Methods

### 2.1. Study Design

This prospective, phase II/III, open-label, multicenter study was conducted at two investigative sites: the University Children’s Hospital “Tiršova” (Belgrade, Serbia) and the Institute for Health Protection of Mother and Child “Dr. Vukan Čupić” (Belgrade, Serbia) as part of the LENA studies. The study received approval from the ethics committees of the respective institutions: the University Children’s Hospital in Belgrade (23 October 2015, No 26/308) and the Institute of Mother and Child Health “Dr. Vukan Cupic” (5 April 2017, No. 8/9). Details of the research protocol have been previously published [29].

The Pediatric Investigational Plan (PIP) received approval from the EMA and the Pediatric Committee (PDCO). The study adhered to the principles of Good Clinical Practice and the Declaration of Helsinki. The parents of all participants were required to provide signed informed consent before inclusion and the privacy rights of the subjects have been observed.

### 2.2. Study Population

This research included HF patients with CHD from birth to 6 years old and those with dilated cardiomyopathy (DCM) from birth to 12 years old enrolled between 2016 and 2018. The current study specifically focused on children up to 6 years of age, considering the impact of age-related differences in swallowing capacity. Patients were enrolled according to the inclusion and exclusion criteria defined in the study protocol, as outlined in the manuscript published by Bajčetić et al. [29]. Children with feeding or swallowing disorders were not excluded by the exclusion criteria of the study; however, none of the children included had these conditions.

Subjects with HF due to CHD or DCM were enrolled and divided into two age cohorts: under 6 months and over 6 months to 6 years. The study included patients previously exposed to ACEI therapy, as well as those whose health condition required it for the first time.

### 2.3. Investigational Medicinal Product

The investigational medicinal products were 2 mm ODMTs containing 0.25 mg or 1 mg of enalapril maleate (Ethicare GmbH, Halternam See, Germany). To support the clinical evaluation of this novel formulation, a study in healthy adults compared the bioavailability of enalapril ODMT (administered as both swallowed and dispersed) with an approved enalapril tablet formulation [30]. The study showed that the administration method of the ODMT, whether swallowed or dispersed, had no significant impact on enalapril bioavailability.

**Figure 1:**
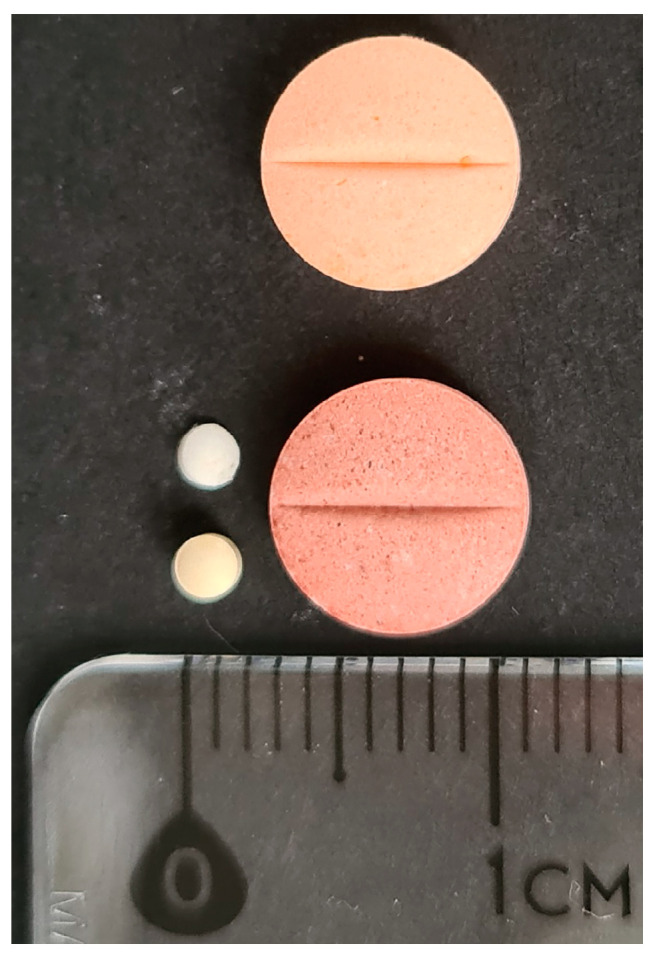
Visual comparison of tablet diameters. Left—top: 2 mm, 0.25 mg enalapril ODMT; bottom: 2 mm, 1 mg enalapril ODMT; Right—top: 8 mm, 10 mg enalapril tablet (adult use); bottom: 8 mm, 20 mg enalapril tablet (adult use).

Figure 1 illustrates the difference in tablet diameters between the ODMT formulation and the conventionally used adult formulation of enalapril.

The maximum tolerated dose of enalapril ODMT was administered following a defined dose-titration scheme or per the investigators’ discretion. Patients received either a single or multiple 0.25 mg ODMT, 1 mg ODMT, or a combination of both, according to the prescribed doses. The ODMTs were administered by placing them in the patient’s cheek pouch, followed by offering a drink of the parents’/patient’s choice (e.g., any type of milk, water, or tea) to facilitate swallowing. The ODMTs were not flavored.

The treatment duration was up to 8 weeks or shorter if the patient could not tolerate the ODMT or if it was no longer medically necessary (with a minimum treatment duration of 3 days). The first dose of enalapril ODMT was consistently administered in a hospital setting, including during dose adjustments at titration visits and during hospital visits scheduled according to the study protocol.

There were no conflicts of interest related to this study and the study was not funded by any industry sponsors.

### 2.4. Study Visits

The study consisted of the following visits:

- Screening Visit (SCRV)—lasting up to 21 days;
- Initiation Visit (IDV)—first ODMT administration;
- Optional Titration Visit(s) (TV)—which could occur up to a maximum of four times;
- Dose Confirmation Visit (DCV);
- Three Study Control Visits (SCV) (at Day 14, Day 28, and Day 42);
- End of Study Visit (EOSV)—Day 56 or the last study visit in case of early termination.

**Figure 2:**
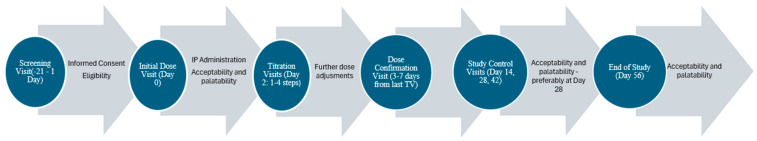
Study visits and assessments overview.

As outlined in Figure 2, acceptability and palatability were assessed at the IDV, during one SCV (preferably Day 28), and at the EOSV. Assessments were conducted after administering the complete dose, which ranged from 1 to 5 ODMTs (0.25 mg and/or 1 mg). For low doses of enalapril (<0.25 mg), the trial protocol allowed using 0.25 mg ODMT enalapril dispersion.

### 2.5. Acceptability and Palatability Assessments—Evaluation Criteria

Acceptability was assessed in children through oral inspection using a short questionnaire method validated by Klingmann et al. depending on the age [21], as outlined in Table 1 and Table 2 [29].

The qualified investigator or appointed representative administered the prescribed dosage form of ODMT(s) into the child’s cheek pouch. After each swallow, the investigator used a torch/flashlight to examine the patient’s mouth, checking for any residual ODMT. A beverage chosen by the parent (such as any type of milk, water or tea) was offered to the child to aid in swallowing. Each swallowing process was closely monitored. The primary outcome measure of acceptability was defined as a combination of two evaluation criteria: “everything swallowed” and “chewed/partially swallowed”.

Palatability assessment was investigated by observing the facial expressions of the children during the 30 s after administration, using the descriptive method outlined in Table 3 [29].

A positive or neutral reaction was classified as palatable.

### 2.6. Statistical Analysis

The analysis included all eligible subjects under 6 years of age who received enalapril ODMT at least at the IDV. Continuous data are presented as mean ± standard deviation (SD), and demographic characteristics are summarized descriptively. A repeated measures ANOVA was conducted to assess whether acceptability and palatability improved with repeated drug administration over three separate visits. Categorical variables were compared using the chi-square test. Statistical significance was set at *p* < 0.05, and all analyses were performed using SPSS version 20.

## 3. Results

### 3.1. Patient Characteristics

A total of 60 subjects were recruited: 38 aged under 6 months and 22 aged 6 months to 6 years. Fifty-three subjects were enrolled with HF due to CHD and 7 subjects had HF due to DCM. In total, 31 individuals were ACEI naïve and 29 patients had a history of ACEI treatment (derived from adult formulations): 23 previously received captopril and 6 had prior enalapril treatment.

The mean (±SD) age of the participants at screening was 0.65 ± 1.04 years, with a mean weight of 6.01 ± 3.65 kg and a mean height of 63.67 ± 13.08 cm. Of the enrolled patients, 45% were male and 55% were female.

The average enalapril ODMT dose was 0.13 mg/kg, with a range of 0.05–0.30 mg/kg. Regarding administered concomitant medications, most patients were treated with furosemide (59 of 60) and spironolactone (57 of 60), while a smaller number received digoxin (7 of 60).

### 3.2. Acceptability and Palatability Assessments

A total of 169 acceptability and palatability assessments were performed. Assessments were conducted on 58 subjects at the IDV. One subject was on dissolved ODMT and assessments were mistakenly overlooked in the other one. There were 59 assessments at the SCV; one was missed due to ODMT discontinuation following hypotension. There were 52 assessments at the EOSV. ODMT was discontinued in six subjects due to cardiac improvement, while it was temporarily halted in one subject due to worsening HF. As previously noted, ODMT was discontinued on one occasion due to hypotension.

As shown in Table 4, acceptability of both enalapril ODMT dosage forms was categorized as either “everything swallowed” or “partially swallowed” in all subjects at all visits. The acceptability as an aggregate of the 2 categories “everything swallowed” and “partially swallowed” was 100%. There were no cases of choking, inhalation/coughing, spitting out or refusal.

Palatability was primarily rated as “pleasant” or “no change”, with six “unpleasant” observations. Since a positive or neutral reaction was classified as palatable, enalapril ODMT was deemed palatable in 96% of cases.

The most frequently prescribed ODMT type was 0.25 mg. In most cases, no beverage was administered. When a beverage was given, it was usually milk or water.

A statistically significant effect was observed for age group differences in ODMT acceptance (Table 5). Children aged 6 months to 6 years showed significantly higher acceptance at both IDV and SCV (*p* < 0.05), with acceptability more frequently classified as “everything swallowed” rather than “partially swallowed”. Additionally, pleasant palatability was reported significantly more often in the older age group at IDV (*p* < 0.05), with this group classifying the ODMT as palatable compared to younger children.

A significant difference in acceptability and palatability was observed between children with HF due to DCM and those with HF secondary to CHD at some visits (*p* < 0.05), with children with DCM demonstrating better acceptance (acceptability more frequently classified as “everything swallowed” rather than “partially swallowed”) and palatability. A significant difference in acceptability and palatability was observed between children who were pretreated with ACEI and those who were ACEI naive across multiple visits as detailed in Table 5, with children pretreated with ACEI demonstrating better acceptability (acceptability more frequently classified as “everything swallowed” rather than “partially swallowed”) and palatability (*p* < 0.05).

At all visits, there was no significant difference between sexes in terms of acceptability and palatability. The same applies to the number and dose of ODMT administered, whether 0.25 mg or 1 mg. Additionally, the co-administration of a beverage had no impact on overall acceptability or palatability. Similarly, no significant difference was observed in acceptability and palatability when compared across the three visits.

## 4. Discussion

This study demonstrated that the acceptability and palatability of both single and multiple enalapril 2 mm ODMTs were favorable among children aged from birth to 6 years diagnosed with HF due to CHD and DCM. To our knowledge, this is the first study to investigate the acceptability and palatability of an ODMT containing an active pharmaceutical ingredient in pediatric patients with chronic diseases requiring repeated administration.

At all study visits, enalapril ODMT was deemed acceptable by patients, as indicated by the aggregate of two criteria: “everything swallowed” and “partially swallowed”. This positive outcome is critical for ensuring patient adherence, which directly impacts the safety and efficacy of the medication. The acceptability rate was not significantly influenced by factors such as repeated administration, dose, number of ODMTs administered, or co-administration of beverages. However, children older than 6 months showed significantly higher acceptability than younger subjects at IDV and SCV, consistent with findings for placebo minitablets in another study [31]. Additionally, acceptability varied significantly between children with HF due to DCM and due to CHD at one visit, which may be due to their older age. A significant difference in acceptability was also noted between children pretreated with ACEIs and ACEI naive at all visits. Prior exposure to enalapril or captopril in the pretreated group may have shaped their responses to the ODMT, suggesting that familiarity with ACEIs could enhance acceptability.

Existing studies on minitablets primarily focus on placebo formulations rather than active pharmaceutical ingredients and lack data on chronic administration. Thomson et al. evaluated the ability of children aged 2–6 years to swallow a 3 mm minitablet, concluding that minitablets could serve as a viable formulation for preschool-aged children [23]. When the swallowability of 2 mm minitablets was compared with 3 mL of syrup in children aged 6 months to 6 years, a significantly higher percentage could swallow the minitablet without chewing, unlike those swallowing syrup, who often left residual liquid [27]. Similarly, Klingmann et al. (2013) demonstrated that a single 2 mm placebo minitablet was significantly more acceptable and easier to swallow than syrup in children aged 6 months to 5 years [24]. When this research was expanded to include neonates (2–28 days), 2 mm minitablets were accepted comparably to syrup, with better swallowing capabilities [25]. Additionally, it was reported that children aged 6 to 23 months found swallowing multiple minitablets easier than consuming dispersed granules or liquid formulations [26]. Collectively, these studies underscore the advantages of minitablet formulations in improving medication acceptability and potentially adherence among young children [32] consistent with our findings regarding the enalapril ODMT. Few data exist on the palatability of enalapril formulations; however, a study using adult volunteers (pediatric medical officers and pediatricians) that assessed various medicinal products indicated that pulverized crushed enalapril 2 mg had a poor taste profile [33,34].

Our study had several limitations. Both acceptability and palatability assessments were conducted in a hospital setting by a designated team member, which may have influenced the results. Additionally, the number of patients included in the study population was relatively small. Future research would greatly benefit from investigating the differences in these two parameters between ODMTs and currently used unlicensed preparations, such as crushed and dispensed tablets and capsules available on the market in Europe.

ODMTs, designed to rapidly disintegrate in the mouth, offer a potential solution for children with swallowing difficulties, gastrointestinal disorders, and those who are tube-fed, thanks to their small size and quick dissolution. However, limited research currently supports the use of ODMTs in children with swallowing or gastrointestinal issues. Golhen et al. emphasized the advantages of ODT-based drug administration for infants and young children, positioning ODMTs as a promising option for pediatric drug delivery [35]. Ejeta further highlighted that ODTs improve drug safety and efficacy, making them suitable for pediatric, geriatric, and dysphagic patients, as they dissolve quickly for easier administration [36].

In addition, ACEI nanoformulations in pediatric patients should be investigated to potentially enhance bioavailability, targeting, and dosing precision, while enabling sustained-release or more palatable options to achieve high adherence [37].

## 5. Conclusions

Our study is the first to investigate the acceptability and palatability of a novel ODMT pediatric formulation for children aged from birth to six years with HF due to CHD or DCM. The results indicated that ODMTs were widely accepted among all children. Additionally, our findings suggest that ODMTs could be a suitable method for administering other medicinal products, particularly in young children. These results support the WHO’s call for a paradigm shift toward small-sized solid dosage forms as an alternative to liquid formulations.

## Figures and Tables

**Table 1:** Evaluation of the acceptability of ODMTs in children aged under 6 months.

Criterion	Definition
1 Everything swallowed	No residue found during oral inspection
2 Partially swallowed	No direct swallowing or residue found during oral inspection
3 Choked on	ODMT was inhaled or caused coughing
4 Termination	The procedure was discontinued on the decision of the assessor, a parent or the patient

**Table 2:** Evaluation of the acceptability of ODMTs in children aged 6 months and older.

Criterion	Definition
1 Everything swallowed	No chewing during deglutition and no residue found during oral inspection
2 Chewed/Partially swallowed	Chewing observed and/or majority of the tablet pieces swallowed, but small residue found during oral inspection
3 Spat out	Spat out
4 Inhaled/coughed	ODMT was inhaled or caused coughing
5 Refused to take	Not allowing the investigator to place ODMT in mouth

**Table 3:** Evaluation of the palatability of ODMTs.

	Criterion	
1	Pleasant	Positive hedonic pattern
2	No change	Neutral
3	Unpleasant	Negative aversive pattern

**Table 4:** Acceptability and palatability results.

	Acceptability		Palatability			ODMT Dose		Beverage	
	Everything swallowed	Partially swallowed	Pleasant	No change	Unpleasant	0.25 mg	1 mg	Yes	No
IDV	32	26	26	28	4	52	6	11	47
SCV	40	19	35	22	2	51	8	6	53
EOSV	39	13	32	20	0	44	8	5	47

**Table 5:** Statistically significant differences in acceptability and palatability by subgroup.

Relation Between:	Chi-Square
Acceptability:	
Age group, IDV	χ^2^ (1, N = 58) = 4.852 *p* = 0.028
Age group, SCV	χ^2^ (1, N = 59) = 4.473 *p* = 0.034
HF etiology, SCV	χ^2^ (1, N = 59) = 3.773 *p* = 0.052 (borderline)
Pretreatment, EOSV	χ^2^ (1, N = 52) = 5.778 *p* = 0.016
2. Palatability:	
Age group, IDV	χ^2^ (1, N = 58) = 4.826 *p* = 0.028
HF etiology, IDV	χ^2^ (1, N = 58) = 7.269 *p* = 0.007
Pretreatment, SCV	χ^2^ (1, N = 59) = 5.207 *p* = 0.022
Pretreatment, EOSV	χ^2^ (1, N = 52) = 13.268 *p* = 0.000

## Data Availability

The data presented in this study are available upon reasonable request from the corresponding author.

## Abbreviations

**Abbreviation**	**Full Term**
ACEIs	Angiotensin-Converting Enzyme Inhibitors
ANOVA	Analysis of Variance
CHD	Congenital Heart Disease
DCM	Dilated Cardiomyopathy
DCV	Dose Confirmation Visit
EMA	European Medicines Agency
EOSV	End of Study Visit
EU	European Union
FSOD	Flexible Solid Oral Dosage
GCP	Good Clinical Practice
HF	Heart Failure
HCP	Healthcare Provider
IDV	Initiation Visit
LENA	Labeling of Enalapril from Neonates up to Adolescents
ODMT	Orodispersible Minitablet
ODT	Orally Disintegrating Tablet
PDCO	Pediatric Committee
PIP	Pediatric Investigational Plan
SCV	Study Control Visit
SCRV	Screening Visit
SD	Standard Deviation
SmPC	Summary of Product Characteristics
SPSS	Statistical Package for the Social Sciences
TV	Titration Visit
WHO	World Health Organization
